# Leucémie à plasmocytes primitive: à propos de 03 cas

**DOI:** 10.11604/pamj.2016.24.167.9791

**Published:** 2016-06-28

**Authors:** Mouhcine Miloudi, Nezha Messaoudi

**Affiliations:** 1 Laboratoire d’Analyses Médicales, 3ème Hôpital Militaire, Laayoune, Maroc; 2 Laboratoire d’Hématologie, Hôpital Militaire d’Instruction Mohammed V, Rabat, Maroc

**Keywords:** Leucémie à plasmocytes, plasmocytose, myélome multiple, Plasma cell leukemia, lasmacytosis, multiple myeloma

## Abstract

La leucémie à plasmocytes est une hémopathie maligne rare définie par la présence de plus de 20 % de plasmocytes de la formule leucocytaire ou un nombre de plasmocytes circulants supérieur à 2 × 10^9^/L (2G/L). Elle peut être primitive, dans 60% des cas, et se manifeste d'emblée sur un mode leucémique ou secondaire, dans 40% des cas, compliquant un myélome multiple connu. Vu la rareté de cette affection, seuls quelques cas ont été rapportés dans la littérature. Elle est caractérisée par son agressivité et son mauvais pronostic. A travers 03 cas diagnostiqués au laboratoire d'hématologie de l'hôpital militaire Mohammed V les auteurs présentent les particularités cliniques, biologiques et pronostic de cette affection.

## Abstract (en)

Plasma cell leukemia is a rare hematologic malignancy defined by the presence of more than 20% of leukocyte count plasma cells or by a number of circulating plasma cells greater than 2 × 10^9^/L (2G/L). It may be primary in 60% of cases and it manifests itself immediately as a leukemia or as a secondary event, in 40% of cases, complicating a previously diagnosed multiple myeloma. Given the rarity of this condition, only a few cases have been reported in the literature. It is characterized by its aggressiveness and poor prognosis. This study reports 3 cases diagnosed in the hematology laboratory of Mohamed V Military Hospital and aims to describe the clinical, biological and prognostic features of this disease.

## Introduction

La leucémie à plasmocytes (LCP) est une prolifération maligne des cellules plasmocytaires définie par une plasmocytose sanguine supérieure à 2 G/L ou un taux de plasmocytes supérieur à 20% de la formule leucocytaire. C'est une prolifération lymphoïde qui représente environ 1 à 3% des leucémies aigues. La forme primitive (LCPp) survient de novo chez un patient non suivi pour myélome multiple (MM), la forme secondaire (LCPs) consiste en la transformation leucémique d'un MM déjà connu. Elle possède des caractères communs avec le MM mais présente également des particularités cliniques, biologiques et pronostiques.

## Patient et observation

**Observation 1:** Patient de 64 ans sans antécédents, le début de sa symptomatologie remontait à 03 mois par l'installation de douleurs rachidiennes inflammatoires sans fièvre dans un contexte d'altération de l'état général. L'examen clinique à l'admission a montré des conjonctives décolorées, le bilan morphologique a objectivé un tassement vertébral en galette de D8. Le bilan pré opératoire a objectivé une insuffisance rénale: urée à 0.62g/l, créatinine à 20mg/l. La numération formule sanguine (NFS) a montré: globules blancs: 9,2gG/l, hémoglobine: 8,9 g/dl, volume globulaire moyen: 101 fl, teneur corpusculaire en hémoglobine: 34pg, plaquettes: 177 G/l, l'étude de frottis sanguin a révélé la présence de 26% de plasmocytes, le myélogramme a objectivé une moelle envahie à 88% par des plasmocytes souvent dystrophiques. Le bilan biochimique a montré une protidémie à 64 g/l, une hypogammaglobulinémie à 4 g/l avec présence de nombreuses bandes d'allure monoclonale à l'éléctrophorèse (EPP), l'immunofixation(IF) a trouvé des chaines légères libres biclonales d'isotype Lambda, le bilan phosphocalcique était normal. La radiographie standard du crâne a objectivé des lacunes à l'emporte-pièce.

**Observation 2:** Patient âgé de 56ans, tabagique chronique, a été admis pour douleur rachidienne type inflammatoire avec toux évoluant dans un contexte d'altération de l'état générale et fièvre. L'examen clinique a trouvé un patient fébrile avec absence de purpura d'hépatosplénomégalie, les aires ganglionnaires étaient libres. La radiographie standard rachidienne a révélé un tassement vertébral au niveau de L1, la radiographie pulmonaire a été en faveur d'une pneumopathie infectieuse. Une NFS a montré une hyperleucocytose à >18,6 G/l avec anémie à 11,8 g/dl normochrome normocytaire et sans thrombopénie, l'étude du frottis sanguin a montré 32% des plasmocytes. L'étude de myélogramme a montré une moelle riche envahie à 42% par des plasmocytes dystrophiques. L'EPP a objectivé une hypogammaglobulinémie, le bilan rénal et phosphocalcique était sans particularités.

**Observation 3:** Patiente de 65 ans, qui présentait depuis plusieurs mois des douleurs lombaires et rebelles aux antalgiques habituels avec une altération progressive de son état général,.un bilan fait en urgence a objectivé une insuffisance rénale. L'hémogramme trouve une hyperleucocytose à 18,6 G/l avec une anémie normochrome normocytaire arégénérative, l'étude de frottis sanguin a mis en évidence une plasmocytose périphérique à 32% faite de plasmocytes souvent dystrophiques. Le taux de protides était de 63 g/l, l'EPP sériques a mis en évidence un aspect oligoclonal dans la zone des gammaglobulines. Le myélogramme a montré une moelle très riche envahie à 65% par des plasmocytes.

## Discussion

**Figure 1:**
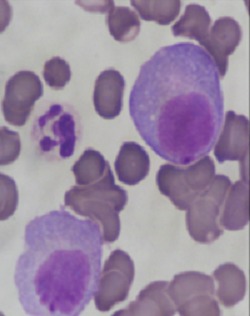
Frottis sanguin coloré au MGG montrant des plasmocytes (objectif 100)

**Figure 2:**
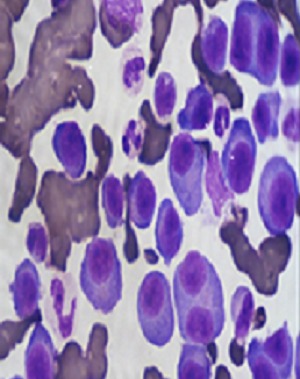
Frottis de la Moelle osseuse coloré au MGG (objectif 100), montrant un envahissement par des plasmocytes souvent immatures et dystrophiques

La LCP est une pathologie rare. Elle correspond à une prolifération plasmocytaire maligne d'origine clonale. Par rapport au MM, la LCP est observée à un âge plus jeune, l´âge médian du diagnostic de la LCP est de 55 ans, alors qu'il est de 65 ans pour le myélome multiple [1]. Les signes cliniques révélateurs sont le plus fréquemment des signes d'insuffisance médullaires. Le tableau clinique est plus agressif que celui du MM avec une plus grande fréquence des atteintes extramédullaires présentes dans 23 à 100 % des cas selon les séries [2], les plus importantes sont les atteintes hépatiques et spléniques retrouvées respectivement dans 52% et 40% des cas de LCPp. Le diagnostic de la LCP est biologique, il repose sur les données de l'hémogramme et le frottis sanguin coloré au MGG (Figure 1) qui montre une plasmocytose sanguine supérieure à 2 G/L ou un taux de plasmocytes circulants supérieur à 20% de la formule leucocytaire. Les plasmocytes sont parfois difficiles à identifier sur frottis sanguins et le recours à l'immunophénotypage dans les formes ambiguës est indispensable au diagnostic. Le bilan est complété par un myélogramme (Figure 2) ou biopsie osteo-médullaire, l'électrophorèse des protéines sériques avec immunofixation, l'électrophorèse des protéines des urines de 24 heures et un bilan biochimique. Par rapport au MM, la LCP est plus fréquemment responsable d'anémies, de thrombopénies, d'hypercalcémies, d'insuffisances rénales et de taux sériques plus élevés de LDH et de ß2-microglobuline (reflet de la masse tumorale). Il est également nécessaire d'effectuer une analyse cytogénétique par la technique d´hybridation fluorescente (FISH) de la population plasmocytaire clonale. La présence d'une translocation (4,14) impliquant le gène codant pour les chaînes lourdes des immunoglobulines ou d'une délétion du 13 est un facteur de mauvais pronostic [3]. Le Tableau 1 compare l'incidence des principales caractéristiques cliniques et biologiques dans LCPp et LCPs. Le traitement de la LCP dépendra de l'âge, du contexte clinique, du bilan d'extension et des paramètres biologiques. Le traitement par Chimiothérapie de type melphalan-prédnisone semble moins efficace (taux de réponse de 20 à 30%, médiane de survie globale de l'ordre de 4 à 8 mois) que les traitements par polychimiothérapie utilisant des combinaisons variables, (exp: protocole VAD ou vincristine, doxorubicine, dexaméthasone) avec un taux de réponse de 40 à 60%, et une survie médiane de 10 à 20 mois. La greffe de moelle après une thérapie d'induction recommandée pour les patients jeunes, a permis d'améliorer d'avantage la survie. Des données récentes préliminaires indiquent que les nouveaux médicaments, en particulier le bortézomib, utilisé en monothérapie ou en combinaison avec d'autres chimiothérapies pourrait améliorer considérablement les résultats cliniques de la LCPp [4, 5].

**Tableau 1:** Incidence des principaux paramètres cliniques et biologiques dans LCPp et LCPs

	LCPp	LCPs
Atteintes extra-médullaires	++++	Moins de 20 %
Douleurs osseuses	25 %	90 %
Lésions lytiques	40-60 %	70%
Infections	35 %	56 %
Plaquettes 100G/L	50 %	71 %
CD28	33 %	92 %

## Conclusion

La leucémie à plasmocytes est une affection rare, de pronostic défavorable. Elle possède des caractères communs avec le MM mais présente également des particularités cliniques, biologiques et pronostiques. La LCP nécessite un traitement intensif qui comprend désormais des combinaisons avec les nouvelles thérapies telles les inhibiteurs du protéasome, et les analogues de la thalidomide. Les intensifications thérapeutiques par autogreffe ou allogreffe chez les patients jeunes ont contribué à l'amélioration du pronostic.

## References

[1] Tiedemann RE, Gonzalez-Paz N, Kyle RA (2008). Genetic aberrations and survival in plasma cell leukemia. Leukemia.

[2] Guieze R, Moreau AS, Dupire S, Coiteux V, Facon T, Leleu X (2005). Leucémie à plasmocytes. Hématologie.

[3] Antony-Debré I, Imbert M (2009). Leucémie à plasmocytes. Revue francophone des laboratoires.

[4] Messaoudia N, Chakour M, El Ktaibib A, Tagjdid R, Belmekki B, Naji N (2009). Leucémie à plasmocytes primitive: une forme rare de leucémie et de prolifération plasmocytaire. Revue francophone des laboratoires.

[5] Chaoui D, Leleu X, Roussel M, Royer B, Rubio M-T, Ducastelle S (2009). Has the prognostic of primary plasma cell leukemia improved with new drugs Blood?. ASH Annual Meeting Abstracts.

